# Influence of dietary n-3 long-chain fatty acids on microbial diversity and composition of sows’ feces, colostrum, milk, and suckling piglets’ feces

**DOI:** 10.3389/fmicb.2022.982712

**Published:** 2022-12-05

**Authors:** Eudald Llauradó-Calero, Eric Climent, Empar Chenoll, Maria Ballester, Ignacio Badiola, Rosil Lizardo, David Torrallardona, Enric Esteve-Garcia, Núria Tous

**Affiliations:** ^1^Animal Nutrition, Institute for Food and Agricultural Research and Technology (IRTA), Tarragona, Spain; ^2^ADM Biopolis, Paterna, Spain; ^3^Animal Breeding and Genetics, Institute for Food and Agricultural Research and Technology (IRTA), Tarragona, Spain; ^4^Animal Health-CReSA, Institute for Food and Agricultural Research and Technology (IRTA), Bellaterra, Spain

**Keywords:** gestating and lactating sows, suckling piglets, n-3 long-chain fatty acids, microbial communities, colostrum, milk, microbial transference

## Abstract

**Introduction:**

Very little is known about the impact of n-3 long-chain fatty acids (n-3 LCFAs) on the microbiota of sows and their piglets. The aim of this study was to evaluate the effect of n-3 LCFA in sow diets on the microbiota composition of sows’ feces, colostrum, and milk as well as that of piglets’ feces.

**Methods:**

Twenty-two sows were randomly assigned to either a control or an n-3 LCFA diet from service to weaning. Sows’ and piglets’ performance was monitored. The gestating and lactating sows’ microbiomes in feces, colostrum, and milk were characterized by 16s ribosomal RNA gene sequencing. The fecal microbiome from the two lowest (>800 g) and the two highest birth weight piglets per litter was also characterized, and the LPS levels in plasma were analyzed at weaning.

**Results and Discussion:**

n-3 LCFA increased microbiota alpha diversity in suckling piglets’ and gestating sows’ feces. However, no effects were observed in colostrum, milk, or lactating sows’ feces. Dietary n-3 LCFA modified the microbiota composition of gestating sows’ feces, milk, and suckling piglets’ feces, without affecting lactating sows’ feces or colostrum. In gestating sows’ feces and milk, the decrease in genus *Succinivibrio* and the increase of Proteobacteria phylum, due to the increased genera *Brenneria* and *Escherichia*, respectively, stand out. In the feces of suckling piglets, the higher abundance of the beneficial genus *Akkermansia* and *Bacteroides*, and different species of *Lactobacillus* are highlighted. In addition, positive correlations for families and genera were found between lactating sows’ feces and milk, milk and suckling piglets’ feces, and lactating sows’ feces and suckling piglets’ feces. To conclude, dietary n-3 LCFA had a positive impact on the microbiome of suckling piglet’s feces by increasing microbial diversity and some beneficial bacteria populations, had a few minor modifications on the microbiome of milk and gestating sows’ feces and did not change the microbiome in lactating sows’ feces or colostrum. Therefore, this study shows the effect of dietary n-3 LCFA on the microbiota of sows, colostrum, milk, and suckling piglets during the lactation period providing crucial information on the microbiota status at the early stages of life, which have an impact on the post-weaning.

## Introduction

The microbiota of pigs is composed of hundreds of different microorganisms and their acquisition and establishment are influenced by different external factors. Concretely, the predominant immediate postnatal factors that determine initial microbial definition in newborn piglets are likely colostrum, milk, feed, oral-fecal transmission, and the neonatal environment (Nowland et al., 2019; Lauridsen, 2020). However, the exact time of initial colonization in pigs has not yet been determined, and some previous reports described prenatal microbial colonization driven by the mother, so it is reasonable to assume that sows’ microbiota would also significantly influence the microbiota of their offspring before or during parturition (Nowland et al., 2021). Moreover, the establishment of the gut microbiota has become a key factor in piglet survival, as suggested by previous studies in humans showing that an appropriate intestinal microbiota resulting from optimal colonization may improve health and limit diseases (Nowland et al., 2019). After all, the microbiota is an important regulator of mammal physiology and could exert several beneficial roles in digestion, protection against pathogens, maintenance of normal function of intestinal villi, and regulation of the immune response (Gresse et al., 2017). In addition, optimal colonization during lactation could become a critical factor considering that the change from maternal milk to solid food during weaning entails microbial modifications that coincide with the morphological and functional maturation of the gut barrier, and with important changes in the gut immune system (Hooper, 2004; Beaumont et al., 2021).

Genetic selection for hyperprolific sows has resulted in a substantial increase in litter size, and this increase in the sows’ prolificacy has resulted in an increased proportion of piglets born with a low weight (< 1.0 kg birth weight) (Quiniou et al., 2002). Low birth weight piglets present a greater energy requirement per kg of body weight (Noblet and Etienne, 1987), and a poorer thermoregulatory ability, which is combined with a limited colostrum intake (Edwards and Baxter, 2015) due to being less vigorous when competing for the limited number of teats in hyperprolific sows. As consequence, these animals present reduced growth rates (Lopez-Verge et al., 2018) and they are less likely to survive. Nutritional strategies applied to the sow may become an effective tool to shape the microbiota establishment of piglets to improve their growth and immune development.

Although under commercial conditions the ingredients in sow diets contain considerable amounts of n-6 long-chain fatty acids (**LCFAs**) and the fat sources used are rich in saturated fatty acids, over the last few years the study of the inclusion of n-3 LCFA sources in sow diets has gained interest (Tanghe and De Smet, 2013). Concretely, research on n-3 LCFA in sow nutrition has mainly focused on their influence on milk composition and offspring performance (Lauridsen, 2020; Llauradó-Calero et al., 2021). Moreover, due to their anti-inflammatory effects (Calder, 2010) and capability to influence the epithelial barrier functions (Liu, 2015), the impact of n-3 LCFA on immune status (Huber et al., 2018) and intestinal epithelium function (Liu et al., 2012) has also been evaluated. However, little is known about the influence of n-3 LCFA on microbiota diversity and composition in sows’ feces, colostrum, milk, and piglets’ feces.

Considering the sow as the first and most relevant factor for the establishment of an optimal microbiota in the newborn piglet, the aim of the current study was to evaluate the inclusion of n-3 LCFA, concretely a solid fish oil rich in eicosapentaenoic acid (**EPA**) (C20:5 n-3) and docosahexaenoic acid (**DHA**) (C22:6 n-3), in sow diets and their impact on the microbiota of gestating and lactating sows’ feces, colostrum, milk and the feces of suckling piglets. The impact of n-3 LCFA on the sow to piglet microbial transference was also analyzed.

## Materials and methods

### Ethics statement

Institute for Food and Agricultural Research and Technology’s (IRTA) Ethical Committee on Animal Experimentation approved the use of animals for this experiment in accordance with Directive 2010/63/EU of 22 September 2010 and according to the recommendation of the European Commission 2007/526/CE, the Spanish guidelines for the care and use of animals in research (B.O.E. number 34, Real Decreto 53/2013) and the regional regulations on the use and handling of experimental animals (Decree 214/97, Generalitat de Catalunya) (project number: 10294).

### Animals, experimental design, and housing

Twenty-two sows in two batches (12 and 10 sows met the selection criteria of being between the third and the sixth parity, respectively) were fed one of the two experimental diets from service until the end of lactation (c.a. 28 days post-farrowing). Within each batch, sows were grouped regarding their body weight and their parity number into pairs, as similar as possible, and sows in each pair were randomly assigned to either a control or an n-3 long-chain fatty acid (n-3 LCFA) diet. At birth, the two piglets with the lowest (>800 g) (LBW) and the two piglets with the heaviest (HBW) body weight in each litter were selected for future microbiome analyses. Cross-fostering of piglets was performed only during the first 24 h of life to standardize litter size to 12 piglets per sow, whenever possible, solely among sows belonging to the same experimental treatment, and without involving selected piglets. Sow feeds were provided *ad libitum* in self-dispensing feeding hoppers. At 11 days of age, piglets were offered either a control or an n-3 LCFA creep feed according to the corresponding maternal diet, using floor-attached round feeders. Water was provided *ad libitum* from nipple drinkers.

Sows were allocated in individual stalls from service until pregnancy confirmation. Once confirmed, they were group-housed in a gestation barn until 1 week before farrowing. Sows were then relocated to individual farrowing crates (0.7 x 2 m) in pens equipped with partially slatted floors and a heated floor panel for piglets (set at 32–34°C). The farrowing room was lit with natural light from a window and with fluorescent artificial light (manually operated), and ventilated *via* single, variable-speed fans linked to temperature sensors. The inside temperature of the building was automatically controlled, and the target temperature of the stables at farrowing was set at 24°C and it was reduced by 0.5°C per week during the lactation period.

Unfortunately, one sow from the second batch (n-3 LCFA treatment) farrowed out of the scheduled time, without supervision, and it was not possible to record the litter characteristics at birth and sample colostrum, so the sow was removed from the trial.

### Experimental diets

Barley–corn-based gestation and lactation diets for sows and the creep feed for piglets were formulated in accordance with FEDNA specifications (de Blas et al., 2013), and their ingredient and nutrient composition were already described by Llauradó-Calero et al. (2021). Diets were formulated to contain the same level of nutrients (metabolizable energy, crude protein, digestive lysine, and ether extract) except for the fat content. In control diets, dietary fat was included using a common animal fat source (5 Sysfeed^®^; Sysfeed SLU, Granollers, Spain) at 15 and 30 g/kg in the gestation and lactation phases, respectively. In the test diets (n-3 LCFA), 15 g/kg of fat was replaced (totally during gestation and one-half during lactation) by solid fish oil (Lipomega^®^; V&S Asociados, Madrid, Spain). Creep feed diets for piglets contained 30 g/kg of animal fat or an equivalent amount of solid fish oil in the control or n-3 LCFA diet, respectively.

Feed intake of sows was restricted to a maximum of 3 kg/day during the gestation period and progressively increased after farrowing until reaching *ad libitum* feed intake. Piglets’ creep feed was offered *ad libitum* from day 11 post-farrowing.

### Growth measurements and sampling

Sows were weighed at service, 1 week before farrowing (when moved to the farrowing crates), the day after farrowing, and at weaning. In addition, backfat thickness in the P2 position of sows was also measured at the same time points (except the day after farrowing) through ultrasound scanning (Piglog 105^®^; Frontmatec, Kolding, Denmark). Daily feed intake was monitored and recorded individually during gestation and lactation. The average daily gain of gestation period was calculated from service to day 107 of gestation (1 week before farrowing), and the average daily gain of lactation period from 1 day after farrowing to weaning (c.a. 28 days post-farrowing). At birth, the total number of piglets born, piglets born alive/dead, mummies, and their individual weights were recorded for each sow. During lactation, the weight of piglets was monitored at 24 h, at 20 days of age, and weaning. Cross-fostering was performed within 24 h after birth and the 24 h recordings were considered as the initial values for litter characteristics and growth performance of suckling piglets during lactation. In the same way, the average daily gain of litters and piglets was calculated from 24 h post-farrowing to each weighing time. Creep feed disappearance was monitored from day 11 of lactation until weaning. One sow from the n-3 LCFA group gave birth to less than six piglets and the corresponding data for litter characteristics at birth (obtained before performing cross-fostering) were excluded from the analysis.

Individual animals were selected for the microbiome studies. Fecal samples from all the sows were collected 1 week before parturition (day 107 of gestation) and at weaning after removing the piglets (c.a. day 28 post-farrowing). At birth, the two lowest (> 800 g) and the two highest birth-weight piglets in each litter were selected and their feces were sampled at weaning. In all cases, samples were individually preserved with Real stock buffer (Durviz, Paterna, Spain) and stored at −80°C until analysis.

Colostrum samples from each sow were obtained immediately after the birth of the first piglet and milk samples were collected at weaning after the piglet’s removal. Sows were milked from all mammary glands following i.v. injection of 1.0 ml of oxytocin (20 IU/ml) (Super’s Diana S.L., Parets del Vallès, Spain). The samples from the different nipples in each sow were pooled and aliquots of at least 3 ml were immediately frozen and stored at −80°C until analysis for microbiome determination.

Blood samples from selected piglets were collected at weaning. Blood was obtained by jugular venipuncture in tubes with ethylenediaminetetraacetic acid (EDTA) and was kept at 4°C a maximum of 120 min until centrifugation (3,000 rpm, 10 min). Plasma aliquots for LPS measurement were obtained and stored at −80°C for a maximum of 30 min after centrifugation.

### Deoxyribonucleic acid extraction and bacterial 16S gene amplification and sequencing

For microbiome analysis, DNA from feces was isolated with the aid of QIAamp Power Fecal Pro DNA Kit (Qiagen, Hilden, Germany), with bead beating and enzymatic lysis steps prior to extraction to avoid bias in DNA purification toward the misrepresentation of gram-positive bacteria. For milk and colostrum, samples were processed with bead beating and enzymatic lysis steps followed by the Blood & Tissue Kit (Qiagen) and further DNA concentration. To evaluate the bacterial composition, massive genome sequencing of the hypervariable region V3–V4 of the bacterial 16s rRNA gene was conducted. Samples were amplified using key-tagged eubacterial primers (Klindworth et al., 2013) and sequenced on a MiSeq Illumina Platform, using a 2 × 300nt paired-end strategy, following Illumina Library preparation and sequencing for metagenomic studies protocol (Zhang et al., 2014; Marcel, 2011).

#### Analysis of sequencing data

The resulting sequences were split considering the barcode introduced during the PCR reaction. PEAR program version 0.9.1 was used to overlap R1 and R2 reads (overlap of 50 nt and quality of the overlap with a minimum of Q20 (Zhang et al., 2014), providing a single FASTQ file for each of the samples. Cutadapt v2.6 (Marcel, 2011) was used to trim 16S rRNA PCR primers and sequences were treated with a quality filter, removing low-quality fragments (under Q20 in Phred scale) and short sequences (under 200nt). Chimeric sequences that potentially arise during the PCR amplification step were identified de novo using CD-HIT software v4.8.1 (Li et al., 2012) and removed. CD-HIT software was also used to create OTUs at 99.7% of identity. The BLAST tool was used to taxonomically identify each OTU against the National Center for Biotechnology Information (NCBI) 16S rRNA database (20th December 2020) using BLASTn version 2.10.0+.

### Lipopolysaccharides measurement in plasma of suckling piglets

The sandwich ELISA kit Porcine Lipopolysaccharides (LPSs) ELISA Kit (MBS269464; MyBioSource, San Diego, CA, USA) was employed according to the manufacturer’s instructions for the quantitative measurement of LPS in all plasma samples from weaned piglets. Intra-assay precision and inter-assay precision of the kit were ≤ 8% and ≤ 12%, respectively.

### Statistical analysis

The analysis of variance of growth and performance data of gestating and lactating sows and suckling piglets and LPS concentration data of plasma from suckling piglets were performed through the GLIMMIX procedure of SAS software (SAS/STAT 14.1; SAS Institute Inc., Cary, NC, USA). For growth and performance measurements, dietary treatment was included in the model as the fixed effect and batch as the random effect. Sow body weight at the beginning of the trial and the parity number were initially introduced into the model as covariates. However, only the initial sow body weight was included in the statistical analysis since parity had no significant effect. In addition, the variable days of lactation were included in the model as a covariate for all the data at weaning. In terms of stillborn piglets, deaths, and mummies, data were normalized using a square root transformation $\left(\sqrt{\text{X}+0.5}\right)$, but the means of the original data are presented in tables. For LPS concentrations, the model included dietary treatment as the fixed effect and sow as the random effect. All data were tested using Kolmogorov–Smirnov test to identify possible outliers and values were excluded if *P* < 0.01. Results were expressed as means ± SD. Significant differences were set at *P* < 0.05.

Microbiome analyses were done using R software (R Core Team, 2012). Alpha and beta diversity indexes were obtained using the vegan package, as implemented for R version 3.2.3 (Oksanen et al., 2020). Bray–Curtis distances were selected to analyze beta diversity, and their significance was studied with PERMANOVA tests. DESeq2 package from R (Love et al., 2014) was used to generate a generalized linear model with fixed effects (control vs. n-3 LCFA diet) with negative binomial family to compare operational taxonomic unit (OTU) counts between groups and select the potential bacterial biomarkers. *P*-values were corrected for multiple testing with Benjamini and Hochberg method, and the statistical significance cutoff was set at *P* < 0.05. The ratios Firmicutes/Bacteroidetes and *Lactobacillus*/Proteobacteria were calculated through relative abundances and adjusted to a maximum value of 100. Higher values were considered outliers and were removed from the analysis. Significant differences were set at *P* < 0.05, while tendencies at *P* < 0.1 using Mann–Whitney–Wilcoxon test.

The PROC CORR procedure of SAS software was used to obtain the Pearson correlation coefficient of the relative abundances of differential microbial populations regardless of the dietary treatment between gestating sows’ feces and colostrum, lactating sows’ feces and milk, milk and sucking piglets’ feces, and lactating sows’ feces and suckling piglets’ feces to study the sow-piglet microbial transference and between suckling piglets’ feces and growth measurements of suckling piglets to identify possible microbial populations affecting the animal growth. A significant correlation level was set at *r* > 0.5 and *P* < 0.05.

## Results

### Sows’ weight, feed Intake, litter characteristics, and piglets’ weight

The body weight, backfat thickness, and feed intake of sows during gestation and lactation are presented in Supplementary Table 1. Sow’s body weight at service (*P* = 0.494), 107 days of gestation (*P* = 0.207), one day after farrowing (*P* = 0.336), and at weaning (*P* = 0.812), and sow’s average daily weight gain during gestation (*P* = 0.202) and lactation (*P* = 0.278) did not differ between dietary treatments. In the same way, backfat thickness in the P2 position at service (*P* = 0.696), 107 days of gestation (*P* = 0.795), and at weaning (*P* = 0.502), and the average daily feed intake during gestation (*P* = 0.787) or lactation (*P* = 0.683) did also not differ either between treatments.

The number of piglets born, piglets born alive/dead, mummies, piglet’s weight, average daily gain during lactation, and creep feed intake are presented in Supplementary Table 2. Litter characteristics and the average piglet’s body weight did not show any significant differences between treatments at birth, 24 h after birth, 20 days after birth, or at weaning (all *P* ≥ 0.166). In the same way, no effects of dietary fish oil were observed for litter or piglet average daily weight gain during the first 20 days (*P* = 0.339 and *P* = 0.553, respectively) or during the whole lactation period (*P* = 0.974 and *P* = 0.714, respectively). Piglet creep feed disappearance did not differ between control and n-3 LCFA diets (*P* = 0.599) either.

### Microbiota diversity

Table 1 shows richness and diversity values for each experimental group and type of sample at phylum, family, genera, and species levels. The largest differences in terms of alpha diversity were observed in the feces of suckling piglets. An increase in microbiota diversity calculated through Simpson and Shannon indices in all studied levels was detected in piglets from the n-3 LCFA sows compared with piglets from the control sows. In addition, microbiota diversity was significantly increased by dietary fish oil through the Simpson index in feces from n-3 LCFA gestating sows.

**TABLE 1:** Microbiota alpha diversity indices, calculated at phylum, family, genera, and species level, of feces of gestating and lactating sows, colostrum, milk, and feces of suckling piglets from animals fed either control or n-3 LCFA diets.^1^

Alpha diversity indices									

	Richness			Simpson index			Shannon index		
			
Taxonomy level	Control	n-3 LCFA	*P*-value	Control	n-3 LCFA	*P*-value	Control	n-3 LCFA	*P*-value
**Gestating sows’ feces**									
Phylum	7.65 ± 0.63	7.83 ± 0.55	0.48	0.57 ± 0.02	0.55 ± 0.02	0.11	1.01 ± 0.08	0.96 ± 0.09	0.21
Family	27.0 ± 1.28	27.1 ± 1.33	0.83	0.74 ± 0.04	0.77 ± 0.04	0.073	1.90 ± 0.10	1.94 ± 0.15	0.47
Genera	50.1 ± 1.44	49.6 ± 2.59	0.55	0.74 ± 0.04	0.77 ± 0.04	0.068	2.08 ± 0.11	2.12 ± 0.19	0.49
Species	80.1 ± 3.14	81.3 ± 4.96	0.52	0.75 ± 0.04	0.79 ± 0.05	0.037	2.31 ± 0.14	2.45 ± 0.25	0.14
**Colostrum**									
Phylum	8.04 ± 0.78	8.19 ± 0.35	0.58	0.54 ± 0.14	0.54 ± 0.08	0.93	1.08 ± 0.24	1.08 ± 0.14	0.94
Family	52.1 ± 4.98	52.8 ± 3.36	0.69	0.82 ± 0.17	0.83 ± 0.09	0.83	2.50 ± 0.53	2.47 ± 0.41	0.91
Genera	102 ± 19.5	102 ± 9.93	0.92	0.84 ± 0.16	0.85 ± 0.10	0.78	2.81 ± 0.60	2.81 ± 0.49	0.99
Species	206 ± 43.8	200 ± 29.4	0.72	0.91 ± 0.07	0.91 ± 0.09	0.99	3.58 ± 0.54	3.58 ± 0.56	0.97
**Lactating sows’ feces**									
Phylum	8.18 ± 0.40	8.06 ± 0.35	0.46	0.59 ± 0.04	0.54 ± 0.07	0.064	1.06 ± 0.10	0.97 ± 0.15	0.091
Family	21.9 ± 0.76	21.9 ± 1.16	0.97	0.75 ± 0.04	0.77 ± 0.03	0.51	1.85 ± 0.13	1.84 ± 0.13	0.80
Genera	48.0 ± 1.77	47.6 ± 2.42	0.61	0.77 ± 0.05	0.78 ± 0.04	0.42	2.10 ± 0.19	2.10 ± 0.13	0.96
Species	76. 4 ± 2.86	77.9 ± 4.23	0.36	0.78 ± 0.05	0.80 ± 0.05	0.34	2.36 ± 0.23	2.38 ± 0.14	0.77
**Milk**									
Phylum	8.00 ±< 0.01	7.82 ± 0.60	0.33	0.56 ± 0.09	0.58 ± 0.11	0.58	1.12 ± 0.17	1.17 ± 0.21	0.57
Family	45.6 ± 3.45	46.6 ± 1.81	0.49	0.86 ± 0.05	0.88 ± 0.04	0.34	2.49 ± 0.16	2.62 ± 0.21	0.11
Genera	97.1 ± 7.81	97.1 ± 7.88	0.99	0.87 ± 0.05	0.89 ± 0.04	0.36	2.80 ± 0.20	2.93 ± 0.24	0.16
Species	208 ± 23.6	207 ± 25.0	0.92	0.90 ± 0.06	0.92 ± 0.04	0.34	3.43 ± 0.29	3.63 ± 0.30	0.14
**Suckling piglet’s feces**									
Phylum	9.83 ± 0.89	9.50 ± 0.74	0.064	0.68 ± 0.06	0.71 ± 0.07	0.036	1.36 ± 0.18	1.46 ± 0.21	0.026
Family	35.8 ± 2.76	35.3 ± 2.77	0.45	0.78 ± 0.07	0.83 ± 0.07	0.006	2.09 ± 0.21	2.28 ± 0.29	0.001
Genera	72.7 ± 5.87	70.0 ± 7.18	0.065	0.81 ± 0.07	0.84 ± 0.06	0.021	2.45 ± 0.23	2.56 ± 0.25	0.054
Species	118 ± 10.5	117 ± 12.9	0.62	0.82 ± 0.07	0.86 ± 0.07	0.009	2.69 ± 0.28	2.89 ± 0.34	0.005

LCFA, long-chain fatty acid. ^1^Values are means ± SD of 11 control and 10 n-3 LCFA samples from sows (gestating and lactating sows’ feces, colostrum, and milk) or 44 control and 40 n-3 LCFA samples from piglets (suckling piglets’ feces).

Alpha-diversity in terms of Richness, Simpson, and Shannon indices in colostrum, lactating sow’s feces, or milk were not significantly affected by fish oil in the sows’ diet (Table 1).

### Microbiota composition and differential microbial communities

**FIGURE 1:**
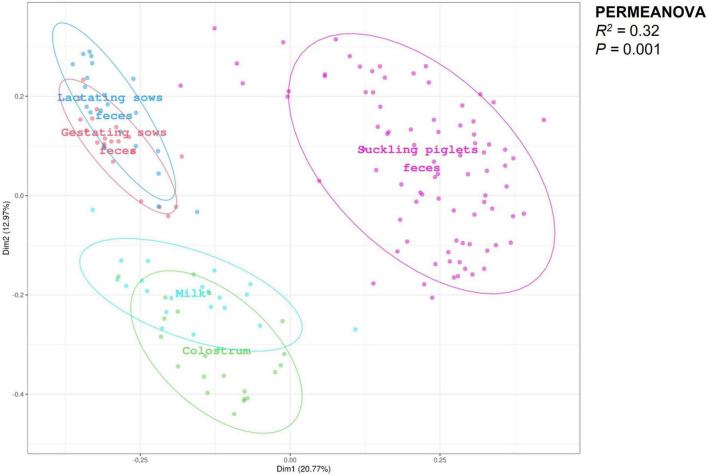
Principal coordinates analysis (PCoA) plot of gestating and lactating sows’ feces (*n* = 21, respectively), colostrum (*n* = 21), milk (*n* = 21), and suckling piglets’ feces (*n* = 84), regardless of experimental dietary treatments, revealed distinct clusters for each sample type (*R*^2^ = 0.32; *P* = 0.001). PERMANOVA test performed using Bray–Curtis distances to analyze beta-dispersion showed differences between all the different types of samples analyzed (all *P* ≤ 0.037) except among gestating and lactating sows’ feces (*P* = 0.235).

The PCoA analysis of sows and piglets’ feces, colostrum, and milk regardless of experimental dietary treatments is presented in Figure 1. PERMANOVA revealed distinct clusters for each sample type (*R*^2^ = 0.32; *P* = 0.001). Beta-dispersion analysis showed differences among all the different types of samples analyzed (all *P* ≤ 0.037) except between feces of gestating and lactating sows (*P* = 0.235).

**FIGURE 2:**
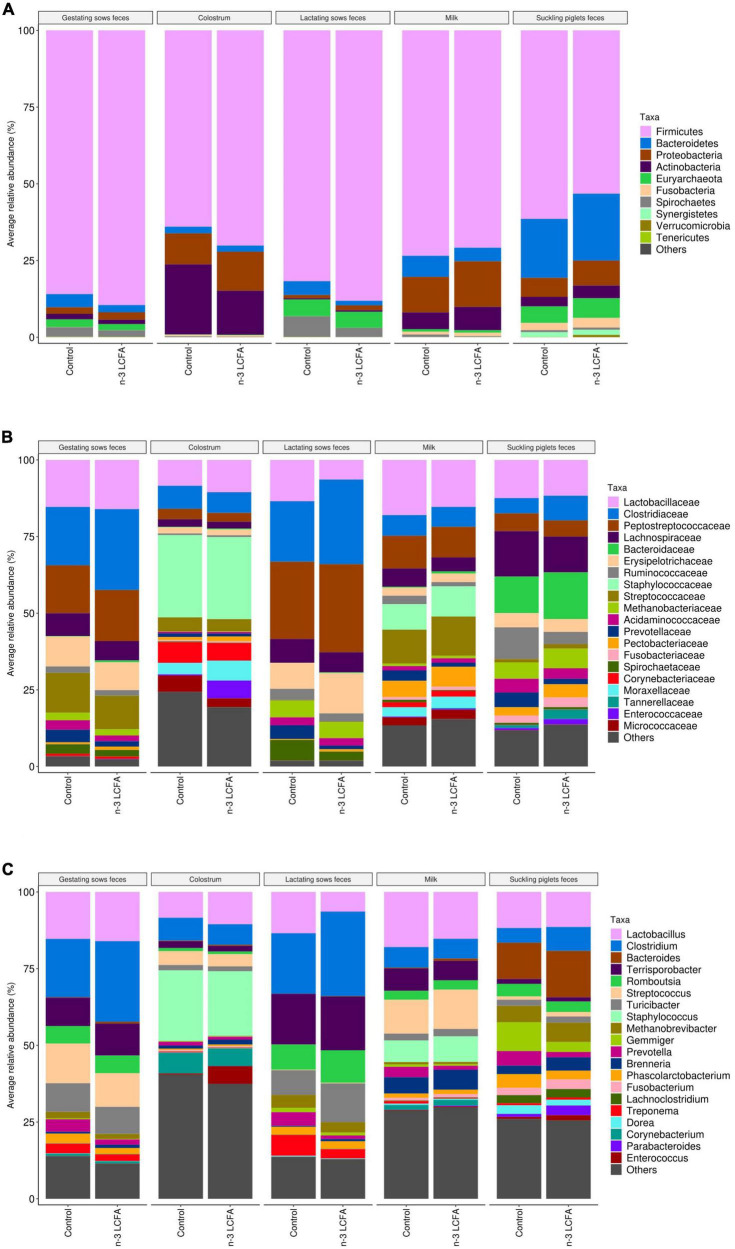
Microbiota composition by the relative abundance of the top 10 phyla **(A)**, top 20 families **(B)**, and top 20 genera **(C)** of feces of gestating and lactating sows (*n* = 21), colostrum (*n* = 21), milk (*n* = 21), and feces of suckling piglets (*n* = 84) according to dietary treatment (control vs. n-3 LCFA). LCFA, long-chain fatty acids.

**FIGURE 3:**
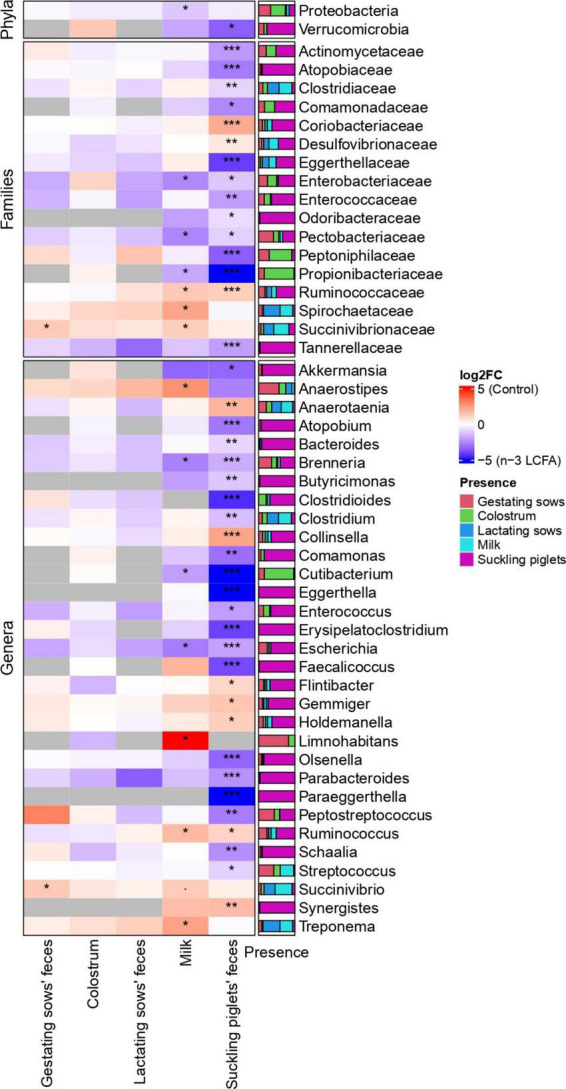
Heatmap representing the differentially abundant phyla, families, and genera between control and n-3 LCFA dietary treatments in feces of gestating and lactating sows, colostrum, milk, and feces of suckling piglets. For feces of gestating and lactating sows, colostrum, and milk; *n* = 11 for control and *n* = 10 for n-3 LCFA. For feces of suckling piglets; n = 44 for control and n = 40 for n-3 LCFA. Significant differences between treatments were set at *P <* 0.05 (*), *P <* 0.01 (**), and *P <* 0.001 (***). LCFA, long-chain fatty acids.

The fecal microbiome of gestating sows was composed mainly of phylum Firmicutes, Families *Clostridiaceae*, *Peptostreptococcaceae*, *Lactobacillaceae*, *Streptococcaceae*, *Erysipelotrichaceae*, and *Lachnospiraceae*, and genera *Clostridium*, *Lactobacillus, Streptococcus, Terrisporobacter*, and *Turicibacter* (Figure 2). Lower abundances of the family *Succinivibrionaceae* (*P* = 0.020) and genus *Succinivibrio* (*P* = 0.031) were observed in the feces of sows from the n-3 LCFA group than those from the control group (Figure 3).

The core microbiome obtained in colostrum samples was represented by phyla Firmicutes, Actinobacteria, and Proteobacteria. *Staphylococcaceae*, *Lactobacillaceae*, *Clostridiaceae*, and *Moraxellaceae* were the dominant Families and *Staphylococcus*, *Lactobacillus*, *Clostridium*, and *Streptococcus* represented the most abundant genera (Figure 2). No differential bacterial communities were detected between control and n-3 LCFA treatments at any taxonomical level studied (Figure 3).

In the feces of lactating sows, Firmicutes was also the most abundant phylum. Families *Peptostreptococcaceae*, *Clostridiaceae*, *Erysipelotrichaceae*, *Lactobacillaceae*, and *Lachnospiraceae* and genera *Clostridium*, *Terrisporobacter*, *Turicibacter*, and *Lactobacillus* had the highest abundance (Figure 2). As for colostrum, no significant differences between the control and n-3 LCFA groups were observed for any taxa at any taxonomical level (Figure 3).

In milk, Firmicutes, Proteobacteria, and Actinobacteria were the dominant bacterial phyla followed by Bacteroidetes. At the family and genus level, the dominant taxa were *Lactobacillaceae*, *Streptococcaceae*, *Peptostreptococcaceae*, *Staphylococcaceae*, and *Lachnospiraceae*, and *Lactobacillus*, *Streptococcus*, *Staphylococcus*, and *Clostridium*, respectively (Figure 2). Differences between the control and the n-3 LCFA treatments were observed for specific groups (Figure 3). Concretely, Phylum Proteobacteria (*P* = 0.017) was increased by dietary n-3 LCFA due to the increased families *Pectobacteriaceae* (*P* = 0.020) and *Enterobacteriaceae* (*P* = 0.020) and the main genera belonging to these families, *Brenneria* (*P* = 0.026) and *Escherichia* (*P* = 0.026), respectively. In addition, other changes by the inclusion of dietary fish oil were detected such as decreases in the family *Ruminococcaceae* (*P* = 0.023) and genera *Ruminococcus* (*P* = 0.026), increases in family *Propionibacteriaceae* (*P* = 0.020) and genera *Cutibacterium* (*P* = 0.026), and decreases in family *Spirochaetaceae* (*P* = 0.023) and genera *Treponema* (*P* = 0.028).

Microbiota composition in the feces of suckling piglets was dominated by phyla Firmicutes, Bacteroidetes, Proteobacteria, Euryarchaeota, and Actinobacteria. *Lachnospiraceae*, *Bacteroidaceae*, *Lactobacillaceae*, *Ruminococcaceae*, and *Clostridiaceae* were the most abundant families, and *Bacteroides*, *Lactobacillus*, *Gemmiger*, *Clostridium*, and *Methanobrevibacter* the most abundant genera (Figure 2). Piglet feces were the type of sample that presented a larger number of differences in microbial communities between treatments. Concretely, one phylum, 15 families, and 27 genera differed between treatments (Figure 3). Phylum Verrucomicrobia (*P* = 0.045) and genera *Akkermansia* (*P* = 0.041) were increased in the feces of piglets from n-3 LCFA-fed sows. In addition, and within Firmicutes, dietary n-3 LCFA reduced the family *Ruminococcaceae* (*P* < 0.001) due to reductions in the genera *Gemmiger* (*P* = 0.032) and *Ruminococcus* (*P* = 0.036). Within the same phylum, the family *Clostridiaceae* (*P* = 0.002) and genera *Clostridium* (*P* = 0.001) were increased by dietary n-3 LCFA. Family *Enterococcaceae* (*P* = 0.010) and genera *Enterococcus* (*P* = 0.011) were also increased by fish oil. It should also be pointed out that the feces from piglets in the n-3 LCFA group had a reduced abundance of genus *Holdemanella* (*P* = 0.032), *Flintibacter* (*P* = 0.024), and *Anaerotaenia* (*P* = 0.009), and an increased abundance of genus *Streptococcus* (*P* = 0.023) and *Erysipelatoclostridium* (*P* < 0.001). Regarding phylum Bacteroidetes, dietary fish oil increased in piglets’ feces in the families *Tannerellaceae* (*P* < 0.001) and *Odoribacteraceae* (*P* = 0.042) due to the increase in their respective genera *Parabacteroides* (*P* < 0.001) and *Butyricimonas* (*P* = 0.002). Within the same phylum also stands out the increased abundance of genera *Bacteroides* (*P* = 0.009) by n-3 LCFA. In terms of phylum Proteobacteria, an increase of families *Pectobacteriaceae* (*P* = 0.047) and *Enterobacteriaceae* (*P* = 0.023), due to the increase in their respective genera *Brenneria* (*P* < 0.001) and *Escherichia* (*P* < 0.001), and a reduction of family *Desulfovibrionaceae* (*P* = 0.006) were observed in piglets from sows fed the n-3 LCFA. In the phylum of Actinobacteria, n-3 LCFA reduced family *Coriobacteriaceae* (*P* < 0.001) and genera *Collinsella* (*P* < 0.001), and increased family *Actinomycetaceae* (*P* < 0.001) and genera *Schaalia* (*P* = 0.007), and family *Atopobiaceae* (*P* < 0.001), including genus *Olsenella* (*P* < 0.001) and *Atopobium* (*P* < 0.001). In addition, n-3 LCFA also reduced genera *Synergistes* (*P* = 0.002) but not modifying phylum *Synergistetes* or family *Synergistaceae*.

**FIGURE 4:**
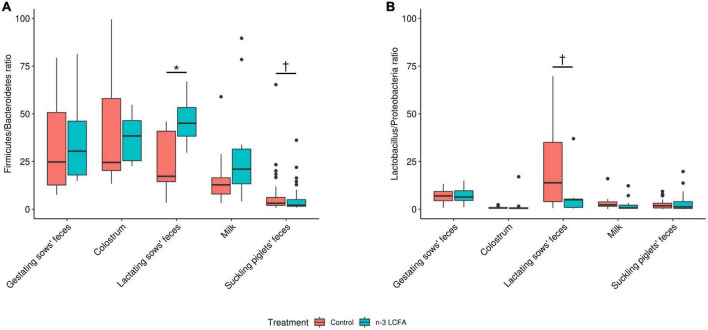
Firmicutes/Bacteroidetes **(A)** and *Lactobacillus*/Proteobacteria **(B)** ratios calculated between treatments in all sample types. For feces of gestating and lactating sows, colostrum, and milk; *n* = 11 for control and *n* = 10 for n-3 LCFA. For feces of suckling piglets; *n* = 44 for control and *n* = 40 for n-3 LCFA. Significant differences between treatments were set at *P <* 0.05 (*), while tendencies were set at *P <* 0.10 (†). Ratios were adjusted at a maximum value of 100 and higher values were removed by being considered outliers. LCFA, long-chain fatty acids.

Finally, the ratios Firmicutes/Bacteroidetes and *Lactobacillus*/Proteobacteria were also compared between treatments for all types of samples, and this is reported in Figure 4. In the feces of lactating sows, an increase in the ratio Firmicutes/Bacteroidetes (*P* = 0.040) and a tendency to decrease the ratio *Lactobacillus*/Proteobacteria were observed (*P* = 0.072) with the n-3 LCFA inclusion, while a tendency to reduce the ratio Firmicutes/Bacteroidetes (*P* = 0.070) was observed in feces of suckling piglets of n-3 LCFA group. For feces of gestating sows, colostrum, and milk, no differences between treatments were observed for either, Firmicutes/Bacteroidetes ratio (all *P* ≥ 0.22) or *Lactobacillus*/Proteobacteria ratio (all *P* ≥ 0.19).

### The concentration of lipopolysaccharides in the plasma of suckling piglets

**FIGURE 5:**
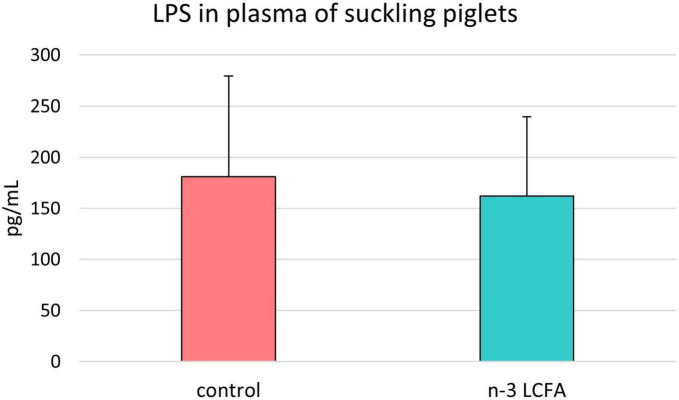
Lipopolysaccharide **(LPS)** concentration in plasma of suckling piglets from control and n-3 LCFA-fed sows. *n* = 44 for control and *n* = 40 for n-3 LCFA. LCFA, long-chain fatty acids.

As shown in Figure 5, no differences in plasma LPS concentration were observed between samples from piglets of the control group and piglets of the n-3 LCFA group (181 ± 98.4 and 162 ± 77.8 pg/ml, respectively; *P* = 0.700).

### Correlations of differential microbial populations between sample types

**FIGURE 6:**
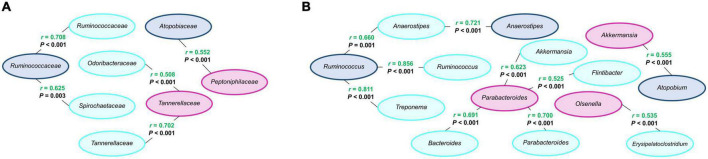
Correlations between modified microbial families **(A)** and genera **(B)** from feces of lactating sows (navy blue, *n* = 21), milk (light blue, *n* = 21), and feces of suckling piglets (purple, *n* = 84). Pearson correlation coefficient (*r*) in green indicates a positive correlation. A significant correlation level was set at *r* > 0.5 and *P* < 0.05.

The comparison of feces from lactating sows and their milk rendered positive correlations at the family level, *Ruminococcaceae* in feces with *Ruminococcaceae* and *Spirochaetaceae* in milk, and four positive correlations at the genus level, *Anaerostipes* in feces with *Anaerostipes* in milk, and *Ruminococcus* in feces with *Anaerostipes*, *Ruminococcus*, and *Treponema* in milk (Figure 6).

Between milk and feces of suckling piglets, two positive correlations were observed at the family level and five positive correlations at the genus level (Figure 6). Concretely, at the family level, *Odoribacteraceae* and *Tannerellaceae* in milk were positively correlated with *Tannerellaceae* in piglets’ feces. At the genus level, *Akkermansia*, *Bacteroides*, *Flintibacter*, and *Parabacteroides* in milk were also positively correlated with *Parabacteroides* in feces and *Erysipelatoclostridium* in milk with *Olsenella* in feces.

One positive correlation at family and genus levels was observed between feces from lactating sows and feces from suckling piglets (Figure 6). Specifically, the correlations were between the family *Atopobiaceae* and genus *Atopobium* in lactating sows’ feces with the family *Peptoniphilaceae* and genus *Akkermansia* in suckling piglets’ feces, respectively.

Finally, no significant correlations between microbial populations were observed at any taxonomical level between gestating sow’s feces and colostrum, and between bacterial populations in suckling piglets’ feces and the growth measurements of suckling piglets during lactation.

## Discussion

The microbiota plays an integral role in influencing host metabolism, its immune system, and the development of a healthy gastrointestinal tract. In addition, the health of offspring is strongly linked to microbial exposure throughout life (Nowland et al., 2019). Considering that weaning is a critical part of pig life characterized by being a complex and stressful event due to the dietary, social and environmental changes (Gresse et al., 2017), the acquisition of an optimal microbiota during the suckling period becomes crucial. Few studies on mice and humans have studied the effect of n-3 fatty acids on the gut microbiota; however, it is described as a poorly understood topic (Costantini et al., 2017). To our knowledge, no prior studies have evaluated the impact of n-3 LCFA on the microbial diversity and composition of sows’ feces, colostrum, and milk and the feces of their suckling piglets, and therefore research on the impact of n-3 LCFA on swine microbiota deserves further attention.

The effects of the inclusion of n-3 LCFA in sows’ diets on sow’s weight, litter characteristics, and growth performance of piglets during lactation have been previously reported (Tanghe and De Smet, 2013). The animals selected for the current study are part of a larger trial studying the impact of n-3 LCFA inclusion in the diets of gestating and lactating diets of sows on different parameters including performance (Llauradó-Calero et al., 2021), and a trend to increase average piglet body weight at weaning is reported, which was in the line with the results of Rooke et al. (2001). However, with the group of sows selected for the current study, no differences in performance between treatments were observed, which would also be consistent with the results of Lauridsen and Danielsen (2004) and Leonard et al. (2010).

Despite diet being one of the main factors affecting intestinal microbiota (Duan et al., 2019), in the current study, the inclusion of fish oil as a source of n-3 LCFA in sow diets cause minor changes in microbial populations in the feces of the sows. This may be due to the fact that sows, as adult animals, are characterized by presenting a stable and well-developed microbiota (Simpson et al., 2000; Niu et al., 2019). However, in gestating sows, microbial diversity increased when they were fed with the n-3 LCFA diet. In addition, the decrease of family *Succinivibrionaceae* due to the decrease of genus *Succinivibrio* stands out, which is considered a core microbiome of the proximal colon or cecum of swine and is related to propionate formation and decarboxylation process (Bergamaschi et al., 2020). However, in lactating sows, no differences in diversity or populations at family and genus levels were found between control and n-3 LCFA-fed animals. Even so, in lactating sows, an increase in the ratio of Firmicutes/Bacteroidetes was observed for the n-3 LCFA group. More Firmicutes and fewer Bacteroidetes have been suggested as a characteristic of fat pigs and have been related to fat deposition (Zhao et al., 2015), although we found no differences between treatments for the weight of the sows or their backfat thickness on P2 position at weaning.

Both colostrum and milk play a critical role in piglets’ development since they are the first sources of nutrients for newborn piglets which are characterized as having low energy reserves and being immunologically naive. In addition, colostrum and milk not only provide nutrients, energy, and immunity, but they also enable the establishment of commensal microbes (Nowland et al., 2019). Given that optimal colostrum and milk supply are crucial for intestinal microbiota colonization and development (Nowland et al., 2021), the impact of n-3 LCFA on colostrum and milk microbiota composition becomes very relevant. In the current study, the bacterial compositions of colostrum and milk were shown to be different, regardless of dietary treatment. On the one hand, the microbiota of colostrum was dominated mainly by phyla Firmicutes, Actinobacteria, and Proteobacteria and families *Staphylococcaceae*, *Lactobacillaceae*, *Clostridiaceae*, and *Moraxellaceae*. On the other hand, the microbiota of milk was dominated by phyla Firmicutes, Proteobacteria, and Actinobacteria and *Lactobacillaceae*, *Streptococcaceae*, *Peptostreptococcaceae*, *Staphylococcaceae*, and *Lachnospiraceae* at the family level. Interestingly, dietary n-3 LCFA impacted differently on the microbial populations from colostrum and milk. In colostrum, no changes were observed in microbial diversity nor differential populations due to n-3 LCFA supplementation. In milk, however, one phylum, six families, and seven genera were modified by the n-3 LCFA diet, although no changes in microbial diversity were observed. Among these changes, the increase in Proteobacteria should be highlighted since it is one of the most dominant phyla in milk microbiota. This increase was mainly due to the higher abundance of families *Pectobacteriaceae* and *Enterobacteriaceae* and the increase in genera *Brenneria* and *Escherichia*, respectively. While no effects of *Brenneria* have been described on animal microbiota, *Escherichia* is a common inhabitant in swine gut microbiota although some of its species could be pathogenic (Schierack et al., 2007). In addition, the decrease of the family *Ruminococcaceae* due to the decrease of the genus *Ruminococcus* stands out. *Ruminococcaceae* has been reported to play a role in the degradation of complex carbohydrates (Crost et al., 2018) and the production of butyrate, and it is associated with anti-inflammatory effects (Liu et al., 2019). Particularly, *Ruminococcus* OTU was identified as *R. flavefaciens*, which plays an important role in the digestion of hemicellulose and cellulose plant cell walls (Fontes and Gilbert, 2010), and their degradation-derived products may act as prebiotics to gut microbiota (Rajan et al., 2021).

Compared to sows, suckling piglets experienced larger modifications in the diversity and bacterial populations of their fecal microbiota due to n-3 LCFA. However, we cannot be sure whether the higher diversity in the fecal samples from n-3 LCFA piglets is due to the direct effect of dietary n-3 LCFA in creep feed, vertical transmission from the sow at birth, or a combination of both. In addition, no effects of birth weight category (low vs. high birth weight piglets) or interactions between dietary treatment and piglets’ birth weight were observed for either diversity or bacterial populations. This contrasts with the results reported by Li et al. (2018), Li N. et al. (2019) who described differences between LBW and normal birth weight piglets during the suckling period for the microbiota of feces and the microbiota of digesta in the ileum and colon. Regarding dietary treatment, bacterial alpha diversity according to Simpson and Shannon indices was increased in suckling piglets on the n-3 LCFA diet. In a previous study, Djuric et al. (2019) described an increase of colonic bacterial diversity in healthy human adults following dietary fish oil supplementation which they related to the anti-inflammatory effects of n-3 LCFA, agreeing with Calder (2019), who suggested a new mechanism by which n-3 LCFA dampen intestinal inflammation. In terms of differential microbial populations, n-3 LCFA modified one phylum, 15 families, and 27 genera in feces from suckling piglets.

Among the observed changes in the fecal microbiota of suckling piglets, the phylum Verrucomicrobia increased due to the increase in the *Akkermansia* genus with its tentative species *A. muciniphila*. A previous study with mice also reported an increase in *A. muciniphila* after fish oil supplementation (Caesar et al., 2015). Moreover, another study in healthy humans described an increase in the family *Akkermansiaceae* in an n-3 LCFA-treated group (Watson et al., 2018). *A. muciniphila* is the most common species of Verrucomicrobia and colonizes the mucus layer acting as a mucin degrader (Ottman et al., 2017). It is well established that it plays a crucial role in supplying mucin-derived nutrients to other members of the gut microbiota that are unable to degrade the mucin layer by themselves (Tailford et al., 2015). This mucin-degrading capacity makes *A. muciniphila* a modulator for gut homeostasis improving and regulating the gut barrier function (Guo et al., 2017). In addition, its presence in the feces of highly feed-efficient animals (Gardiner et al., 2020) and its possible role as the host immune system modulator (Crespo-Piazuelo et al., 2019) have also been previously described. Other studies propose that excessive mucin degradation may facilitate the access of pathogens to the mucosa (Ganesh et al., 2013). However, in the current study, no differences in LPS concentration in the plasma of suckling piglets at weaning, as a gut barrier integrity marker, were observed between n-3 LCFA and control diets.

Within the important bacterial populations of the Firmicutes phylum that were modified by n-3 LCFA in the feces of suckling piglets, we observed a decrease in the family *Ruminococcaceae* and the increase of family *Clostridiaceae*, which are two of the core families in the swine gastrointestinal tract (Holman et al., 2017). Decreased *Ruminococcaceae* was due to the decrease of genera *Ruminococcus* (tentatively identified as *R. gnavus*) and *Gemmiger*, and increased *Clostridiaceae* due to the increase of the genus *Clostridium* and, specifically, the increases of OTUs identified as *C. innocuum*, *C. cadaveris*, and *C. perfringens*. On the one hand, as already described, *Ruminococcaceae* is associated with anti-inflammatory effects (Liu et al., 2019). Conversely, *R. gnavus* has been associated with a pro-inflammatory role (Hall et al., 2017; Henke et al., 2019). On the other hand, members of the family *Clostridiaceae* are also associated with the production of butyrate, so they can also contribute to decreasing inflammation in the gut of the host (Holman et al., 2017). However, species *C. innocuum*, *C. cadaveris*, and *C. perfringens* can become pathogenic causing infections (Crum-Cianflone, 2009; Gupta et al., 2020; Posthaus et al., 2020). Concretely, *C. perfringens* can cause severe, acute, and necrotic enteritis in humans and livestock, particularly in neonatal pigs (Posthaus et al., 2020). The pathogenicity of *C. perfringens* is given by toxin-α and toxin-β, which are generated by *C. perfringens* type A and C (Baker et al., 2010; Posthaus et al., 2020). Although *C. perfringens* infection causes diarrhea, low weaning weights, and pre-weaning mortality, no differences in body weight nor mortality were observed between treatments for suckling piglets at weaning. Within the same phylum, we also observed an increase in the abundance of *Streptococcus*, which contains some species described as probiotics and has been related to the improvement of colostrum quality, milk quality and quantity, litter size, and piglet vitality, and body weight (Knecht et al., 2020). In addition, *Lactobacillus* species (OTUs tentatively identified as *L. delbrueckii* and *Lactobacillus mucosae*) were also increased in the feces of n-3 LCFA suckling piglets. Previous reports have described an immunomodulatory effect of *L. mucosae* (Ryan et al., 2019), and that the oral administration of *L. delbrueckii* improves intestinal integrity, stimulates the intestinal immune response, and alleviates intestinal oxidative damage in piglets (Li Y. et al., 2019; Chen et al., 2020). Moreover, Yang et al. (2017) described that many beneficial bacteria belonging to the Firmicutes phylum, such as *Enterococcus*, *Streptococcus*, *Lactobacillus*, and *Clostridium*, were reduced in diarrheic piglets. In the current study, these beneficial genera and pertaining species were increased by dietary n-3 LCFA.

Relevant modifications of bacterial populations belonging to phyla Bacteroidetes and Proteobacteria by dietary n-3 LCFA should also be noted in the feces suckling piglets. Regarding Bacteroidetes, n-3 LCFA increased the *Bacteroides* genus, which is one of the core bacterial genera of pigs’ microbiota and is reported to be found in more than 90% of healthy pigs of different ages (Luo et al., 2022). Moreover, as described above for *A. muciniphila*, genus *Bacteroides* is also considered to be a mucin glycan degrader (Bell and Juge, 2021) and its low abundance has been associated with post-weaning diarrhea (Ren et al., 2022). It should be mentioned that n-3 LCFA also decreased *Prevotella copri* species, which are present during lactation at low abundances but increase drastically upon weaning (Amat et al., 2020). A high abundance of *P. copri* has recently been related to hosting chronic inflammation responses resulting in excessive fat accumulation in pigs (Chen et al., 2021). In terms of Proteobacteria, n-3 LCFA increased the *Pectobacteriaceae* and *Enterobacteriaceae* families mainly due to the increases of genus *Brenneria* and *Escherichia*, respectively, modifications that match those observed in milk. *Enterobacteriaceae* consists of a set of genera that colonize the intestinal microbiota and includes commensal microbiota as well as pathogens (Schierack et al., 2007). This family contains LPS-producing bacteria and *Escherichia coli* species, which can cause diarrhea and infections in both humans and animals (Schierack et al., 2007; Costantini et al., 2017). However, in the present study, despite *Escherichia* being increased in the feces of n-3 LCFA suckling piglets, no increase in *E. coli* was detected and as already mentioned, there were no differences in piglets’ LPS plasma levels.

According to Lauridsen (2020), colostrum, milk and oral-fecal transmission play a crucial role in the acquisition and establishment of the microbiota of newborn piglets. Some correlations were observed between lactating sows’ feces and milk, milk and suckling piglets’ feces, and lactating sows’ feces and suckling piglets’ feces showing that the changes observed in the former due to the inclusion of dietary n-3 LCFA may be transferred and have an impact on the latter. First, between lactating sows’ feces and milk, the family *Ruminococcaceae* and genus *Ruminococcus* in lactating sows were positively correlated with the same family and genus in milk, but also with the family *Spirochaetaceae*, and genera *Anaerostipes* and *Treponema*. Second, the genera *Akkermansia*, *Bacteroides*, *Flintibacter*, and *Parabacteroides* in milk correlate positively with the genus *Parabacteroides* in suckling piglets’ feces. Concretely, different OTUs belonging to these four previous genera in milk were positively correlated with *Parabacteroides distasonis* in suckling piglets’ feces, which is described as immunomodulatory health-promoting bacteria (Kverka et al., 2011). Moreover, positive correlations between lactating sows’ feces and suckling piglets’ feces were also observed. OTUs such as *Olsenella scatoligenes*, *Erysipelatoclostridium ramosum*, *Peptostreptococcus stomatis*, *A. muciniphila*, and *L. delbrueckii* on feces from lactating sows were positively correlated with the *Bacteroides* species *Bacteroides stercoris* and *Bacteroides fluxus* in feces from suckling piglets. Correlations between species are reported in Supplementary Figure 1. Therefore, milk appears to be the type of sample with more bacterial populations positively correlated with those in the feces of lactating sows or suckling piglets, suggesting its key role in microbial transfer from sow to piglets. Finally, it is important to mention that the study of correlations was performed using samples that were collected at the same time and although the transference of microbiota is not an immediate process, sampling was carried out when both, sows and piglets, had been eating for several days the same diet and it could be assumed that results are representative of the correlations among the different types of samples. Nevertheless, a future study evaluating microbial transference considering a time window between samplings may be of interest.

## Conclusion

The inclusion of fish oil as a source of n-3 LCFA in sow diets influences the microbiota of the feces of gestating sows, their milk, and the feces of suckling piglets, while no effects were observed in colostrum or the feces of lactating sows. The largest impact of n-3 LCFA supplementation is observed in the feces of suckling piglets, which are young animals that are in the process of acquiring microbiota. Concretely, n-3 LCFA increased piglets’ fecal microbial diversity and the relative abundance of beneficial bacteria such as the mucin-degraders genera *Akkermansia* and *Bacteroides*, and different species of *Lactobacillus*, which may contribute to the achievement of a gut anti-inflammatory microbiota. In addition, it can also be concluded that some of these modifications were positively correlated among the feces of lactating sows, their milk, and the feces of the suckling piglets. Milk stands out as the factor with more bacterial populations correlated with both, lactating sows’ and suckling piglets’ feces.

## Data availability statement

The datasets presented in this study can be found in online repositories. The names of the repository/repositories and accession number(s) can be found below: https://www.ebi.ac.uk/ena, PRJEB53326.

## Ethics statement

This animal study was reviewed and approved by IRTA’s Ethical Committee on Animal Experimentation and Generalitat de Catalunya (project no: 10294).

## Author contributions

IB, RL, DT, EE-G, and NT contributed to conception and design of the study. EL-C, ErC, EmC, MB, and NT performed the methodology and statistical analysis. EL-C wrote the first draft of the manuscript. ErC and EmC wrote sections of the manuscript. All authors contributed to manuscript revision, read, and approved the submitted version.
