# Supplementary material for: Influence of dietary n-3 long-chain fatty acids on microbial diversity and composition of sows’ feces, colostrum, milk, and suckling piglets’ feces
Source: Front Microbiol. 2022 Dec 5;13:982712. doi: 10.3389/fmicb.2022.982712 (PMC9760940; doi:10.3389/fmicb.2022.982712)
Supplement: Supplementary file 1 [file Data_Sheet_1.docx]

Supplementary Material

**SUPPLEMENTARY TABLE 1:** Influence of dietary fish oil rich in n-3 LCFA on the body weight, backfat thickness, and feed intake of gestating and lactating sows^1^

|  | Control (*n*=11) | n-3 LCFA (*n*=10) | | | | *P* value | |
| --- | --- | --- | --- | --- | --- | --- | --- |
| Days of gestation | 116 ± 1.10 | 116 ± 1.23 | | | | 0.531 | |
| Days of lactation | 25.1 ± 0.94 | 24.9 ± 1.10 | | | | 0.929 | |
| Average BW (kg) |  | |  |  |  | |  |
| Service | 212 ± 31.0 | 221 ± 28.0 | | | | 0.494 | |
| End of gestation (day 107 of gestation) | 283 ± 23.5 | 286 ± 27.7 | | | | 0.207 | |
| Day after farrowing | 259 ± 27.2 | 271 ± 24.7 | | | | 0.336 | |
| At weaning (c.a. 28 days post-farrowing) | 238 ± 29.5 | 247 ± 25.0 | | | | 0.812 | |
| Average backfat thickness in P2 (mm) |  |  | | | |  | |
| Service | 15.4 ± 4.51 | 15.3 ± 5.13 | | | | 0.696 | |
| End of gestation (day 107 of gestation) | 15.1 ± 4.37 | 15.0 ± 4.47 | | | | 0.795 | |
| At weaning (c.a. 28 days post-farrowing) | 11.3 ± 3.45 | 12.7 ± 4.31 | | | | 0.502 | |
| Average daily gain (kg) |  |  | | | |  | |
| Gestation | 0.62 ± 0.11 | 0.55 ± 0.06 | | | | 0.202 | |
| Lactation | -0.83 ± 0.36 | -1.02 ± 0.36 | | | | 0.278 | |
| Total | 0.19 ± 0.11 | 0.18 ± 0.10 | | | | 0.818 | |
| Average daily feed intake (kg) |  | |  |  |  | |  |
| Gestation | 2.78 ± 0.02 | 2.79 ± 0.03 | | | | 0.787 | |
| Lactation | 5.62 ± 1.05 | 5.45 ± 0.71 | | | | 0.683 | |

BW, body weight; LCFA, long chain fatty acid.

^1^Values are means ± SD.

SUPPLEMENTARY TABLE 2: Influence of dietary fish oil rich in n-3 LCFA on the litter characteristics at birth and growth performance of suckling piglets^1^

|  | Control (*n*=11) | n-3 LCFA (*n*=10) | *P* value |
| --- | --- | --- | --- |
| At birth^2^ |  |  |  |
| Average total born | 15.2 ± 3.82 | 15.4 ± 3.50 | 0.908 |
| Born alive | 14.7 ± 3.41 | 14.8 ± 3.11 | 0.984 |
| Stillborn | 0.46 ± 0.69 | 0.67 ± 0.87 | 0.573 |
| Mummies | 0.55 ± 0.69 | 0.22 ± 0.44 | 0.166 |
| Average litter weight (kg) | 19.7 ± 3.13 | 19.6 ± 3.66 | 0.898 |
| Average piglet BW (kg) | 1.39 ± 0.28 | 1.34 ± 0.19 | 0.665 |
| SD piglet BW (kg) | 0.25 ± 0.05 | 0.25 ± 0.09 | 0.850 |
| 24h after birth^3^ |  |  |  |
| Average still alive | 13.7 ± 1.49 | 12.8 ± 1.23 | 0.169 |
| Average of deaths 24h | 0.64 ± 1.03 | 1.00 ± 1.63 | 0.388 |
| Average litter weight (kg) | 19.4 ± 2.89 | 19.1 ± 2.89 | 0.898 |
| Average piglet BW (kg) | 1.43 ± 0.25 | 1.50 ± 0.20 | 0.476 |
| SD piglet BW (kg) | 0.31 ± 0.05 | 0.29 ± 0.09 | 0.238 |
| 20 days after birth |  |  |  |
| Average still alive | 12.0 ± 1.10 | 11.7 ± 1.25 | 0.752 |
| Average litter weight (kg) | 74.1 ± 9.82 | 74.0 ± 7.04 | 0.785 |
| Litter average daily gain (24h → 20d) (kg) | 2.73 ± 0.40 | 2.80 ± 0.30 | 0.339 |
| Average piglet BW (kg) | 6.19 ± 0.72 | 6.34 ± 0.49 | 0.563 |
| SD piglet BW (kg) | 1.23 ± 0.31 | 1.66 ± 0.53 | 0.879 |
| Piglet average daily gain (24h → 20d) (kg) | 0.25 ± 0.03 | 0.26 ± 0.03 | 0.553 |
| At weaning (c.a. 28 days post-farrowing) |  |  |  |
| Average still alive | 11.9 ± 1.14 | 11.7 ± 1.25 | 0.945 |
| Average of deaths lactation | 1.82 ± 1.54 | 1.10 ± 0.99 | 0.920 |
| Average litter weight (kg) | 95.6 ± 11.2 | 95.0 ± 11.4 | 0.608 |
| Litter average daily gain (24h → W) (kg) | 2.83 ± 0.39 | 2.84 ± 0.31 | 0.974 |
| Average piglet BW (kg) | 8.07 ± 0.98 | 8.13 ± 0.62 | 0.738 |
| SD piglet BW (kg) | 1.51 ± 0.38 | 1.66 ± 0.53 | 0.482 |
| Piglet average daily gain (24h → W) (kg) | 0.25 ± 0.03 | 0.25 ± 0.02 | 0.714 |
| Piglet creep feed intake (kg) | 0.33 ± 0.06 | 0.30 ± 0.06 | 0.599 |

BW, body weight; LCFA, long chain fatty acid; SD, standard deviation; W, weaning.

^1^Values are means ± SD.

^2^One sow from n-3 LCFA diet gave birth less than 6 piglets and were excluded for the data analysis of litter characteristics at birth.

^3^Adoptions were completed within 24 hours after birth and the 24 h recordings were considered as the initial values for the litter characteristics and growth performance of suckling piglets.


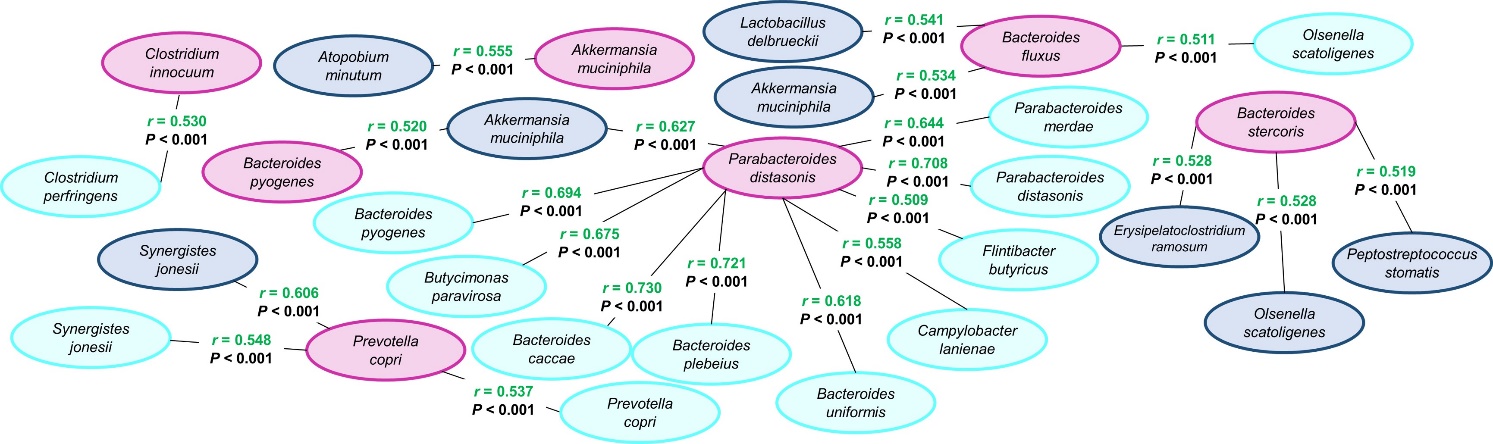


**Supplementary Figure 1.** Correlations between modified microbial species from lactating sows’ feces (navy blue, *n* = 21), milk (light blue, *n* = 21) and suckling piglets’ feces (pink, *n* = 84). Pearson correlation coefficient (*r*) in green indicates positive correlation. Significant correlation level was set at *r* > 0.5 and *P* < 0.05.
